# Utilization of Ocrelizumab within Different Treatment Strategies for Multiple Sclerosis: A 5-Year Population-Based Study

**DOI:** 10.3390/neurolint16020029

**Published:** 2024-03-29

**Authors:** Marcello Moccia, Giuseppina Affinito, Giuseppina Marrazzo, Tiziana Ciarambino, Paolo Di Procolo, Licia Confalonieri, Antonio Carotenuto, Maria Petracca, Roberta Lanzillo, Maria Triassi, Vincenzo Brescia Morra, Raffaele Palladino

**Affiliations:** 1 Multiple Sclerosis Unit, Policlinico Federico II University Hospital, Via Sergio Pansini 5, 80131 Naples, Italy; carotenuto.antonio87@gmail.com (A.C.); roberta.lanzillo@unina.it (R.L.); vincenzo.bresciamorra2@unina.it (V.B.M.); 2 Department of Molecular Medicine and Medical Biotechnology, University of Naples Federico II, 80131 Naples, Italy; 3 Department of Public Health, University of Naples Federico II, 80131 Naples, Italy; giuseppinaaffinito1992@gmail.com (G.A.); triassi@unina.it (M.T.); raffaele.palladino@unina.it (R.P.); 4 ROCHE Spa, 20900 Monza, Italy; giuseppina.marrazzo@roche.com (G.M.); paolo.di_procolo@roche.com (P.D.P.); licia.confalonieri@roche.com (L.C.); 5 General Directorate for Healthcare Protection and Management of the Regional Healthcare Service, Strategic Management Office, Caserta Healthcare Authority, 81100 Caserta, Italy; tiziana.ciarambino@gmail.com; 6 Department of Neuroscience, Reproductive Science and Odontostomatology, University of Naples Federico II, 80131 Naples, Italy; 7 Department of Human Neurosciences, Sapienza University, 00185 Rome, Italy; maria@petraccas.it; 8 Department of Primary Care and Public Health, Imperial College, London W6 8RP, UK

**Keywords:** multiple sclerosis, ocrelizumab, treatment, persistence, adherence, costs

## Abstract

Background: We aim to provide up-to-date real-world evidence on the persistence, adherence, healthcare resource utilization, and costs of multiple sclerosis (MS) by comparing ocrelizumab to other disease-modifying treatments (DMTs) and within different DMT sequences. Methods: We included 3371 people with MS who first received or switched DMT prescriptions from January 2018 to December 2022; they were identified through hospital discharge records, drug prescriptions, and exemption codes from the Campania Region (South Italy). We calculated persistence (time from the first prescription to discontinuation or switching to another DMT), adherence (proportion of days covered (PDC)), DMT costs, and MS hospital admissions and related costs. Results: The most frequently prescribed DMT was dimethyl fumarate (n = 815; age 38.90 ± 11.91 years; 69.5% females), followed by ocrelizumab (n = 682; age 46.46 ± 11.29 years; 56.3%); 28.8% of the patients treated with ocrelizumab were naïve to DMTs. Using ocrelizumab as a statistical reference, the risk of discontinuation was higher for other highly active (HR = 6.32; 95%CI = 3.16, 12.63; *p* < 0.01) and low-/medium-efficacy DMTs (HR = 10.10; 95%CI = 5.10, 19.77; *p* < 0.01); adherence was lower for other highly active DMTs (Coeff = −0.07; 95%CI = −0.10, −0.04; *p* < 0.01) and low-/medium-efficacy DMTs (Coeff = −0.16; 95%CI = −0.19, −0.14; *p* < 0.01). monthly DMT costs were higher for other highly active DMTs (Coeff = 77.45; 95%CI = 29.36, 125.53; *p* < 0.01) but lower for low-/medium-efficacy DMTs (Coeff = −772.31; 95%CI = −816.95, −727.66; *p* < 0.01). The hospital admissions and related costs of MS were similar between ocrelizumab, other highly active DMTs, and other low-/medium-efficacy DMTs, and with ocrelizumab as the first-line DMT after other highly active DMTs and after low-/medium-efficacy DMTs, which was possibly due to the low number of observations. Conclusions: From 2018 to 2022, ocrelizumab was among the most frequently prescribed DMTs, with 28.8% prescriptions to incident MS patients, confirming its relevance in clinical practice. Ocrelizumab was associated with the highest persistence and adherence, pointing towards its favorable benefit–risk profile. The costs of ocrelizumab were lower than those of other highly active DMTs.

## 1. Introduction

The treatment scenario for multiple sclerosis (MS) is characterized by the availability of a number of disease-modifying therapies (DMTs) that can be grossly differentiated by their efficacy (low/medium and highly active) and mode of administration (injectable, oral, and infusion) [1,2]. Differences among DMTs are mostly derived from randomized controlled trials (RCTs), which, however, are based on simple comparisons (new DMTs vs. reference DMTs), a short follow-up (24 months), and highly selected population [1]. Clinical registries have contributed to the evaluation of the comparative and long-term effectiveness of DMTs in the real world [3,4,5], but they are at risk of bias due to patient selection (e.g., the inclusion of patients and clinical variables only from participating centers) and follow-up (e.g., variable follow-up duration, with patients doing poorly being likely to be lost during follow-up) [6,7]. Also, both RCTs and registry-based studies do not consider healthcare resource utilization and, more generally, the complexity of MS management [3]. Not least, the impact of the use of different DMT sequences is largely unexplored, especially in relation to the subsequent burden for healthcare systems.

To overcome these limitations, we have used an algorithm based on routinely collected healthcare data to identify individuals with a diagnosis of MS living in the Campania Region of Italy [8,9], where specific measures of the effectiveness and economic viability of DMTs could be computed. For instance, in a recent study conducted from 2018 to 2020, we showed higher persistence for ocrelizumab when compared with other DMTs, along with a more favorable profile of associated healthcare resource utilization and costs [2]. Hereby, we aim to confirm our previous data on the persistence, adherence, healthcare resource utilization, and direct healthcare costs and to compare these measures between ocrelizumab and other DMTs, as well as within different sequences of DMTs.

## 2. Methods

### 2.1. Study Design

This is a population-based study that was conducted through the retrospective analysis of routinely collected healthcare data, which were prospectively recorded from 2018 to 2022, on individuals with a diagnosis of MS living in the Campania Region of Italy. The original dataset has been described elsewhere [8,9]. For the purposes of the present study, we selected the time frame of 2018–2022 to include ocrelizumab-treated patients from the beginning of its use in the real world (the first prescription was recorded on 6 November 2018) [2].

The study was approved by the Federico II Ethics Committee (332/21). All patients gave their informed consent authorizing the use of anonymized data that were collected routinely as part of clinical practice in line with data protection regulations (GDPR EU2016/679). The study was performed in accordance with good clinical practices and the Declaration of Helsinki.

### 2.2. Study Population

The dataset was created by merging different data sources from the Campania Region [8,9]. In particular, the MS cohort included all individuals who had at least one record in the hospital discharge record database (which included all admissions in the study period with an ICD-9 CM code of MS as one of the discharge diagnoses), the regional drug prescription database (which included all MS-specific DMTs prescribed in the study period), and/or the outpatient database (which included all outpatient consultations with an MS-specific exemption from co-payment records).

The case-finding algorithm had 99.0% sensitivity in the identification of prevalent individuals with MS, with a very low risk of missing individuals (2.7%) [8], and it had 95.3% specificity in the identification of incident individuals with MS [9] when using MS diagnoses with the 2017 revisions of the McDonald criteria as a reference [10]. For the purposes of the present study, we referred to both individual patients and individual treatment periods (ITPs), since the same patient could have used different DMTs during the study period.

Healthcare services (e.g., DMT prescription, inpatient, outpatient) delivered in any part of Italy to individuals resident in the Campania Region are routinely reported to the Campania Region Healthcare Regulatory Society (So.Re.Sa.) for refund purposes [2]. The same processing of routinely collected healthcare data is required for both public and private healthcare facilities. As such, healthcare resource utilization for individuals with MS living in the Campania Region is entirely traceable through the So.Re.Sa.

The inclusion criteria for ITPs were the following: (1) commencing on a DMT during the time frame of 1 January 2018–31 December 2022, which included either switching from a previous DMT or commencing a DMT in the absence of previous treatment records (data from 2015 to 2017 were used as the pre-index period); (2) repeated DMT prescriptions over a minimum follow-up of 3 months (i.e., corresponding to three monthly refills of injectable and oral DMTs, 1 year of cladribine and alemtuzumab dosing, 3 infusions of natalizumab, or 2 loading doses of ocrelizumab). The exclusion criteria were the following: (1) ITPs already including a DMT at the start date (1 January 2018); (2) incomplete records; (3) lack of written consent to participate in the study; (4) residence outside of the Campania Region.

### 2.3. Treatment Variables

DMT prescriptions were collected, and the following DMT groups were defined:

- DMT administration: infusion (alemtuzumab, natalizumab), oral (cladribine, fingolimod, teriflunomide, dimethyl fumarate), and injection (glatiramer acetate, interferon beta-1a, interferon beta-1b, and peg-interferon beta-1a), using ocrelizumab as a reference for comparison [2];
- DMT efficacy: low-/medium-efficacy DMTs (teriflunomide, dimethyl fumarate, glatiramer acetate, interferon beta-1a, interferon beta-1b, and peg-interferon beta-1a) and highly active DMTs (alemtuzumab, natalizumab, cladribine, fingolimod), using ocrelizumab as a reference for comparison [2];
- Previous DMT: ocrelizumab as a first-line DMT (no MS records in the previous 12 months) [9] after low-/medium-efficacy DMTs (teriflunomide, dimethyl fumarate, glatiramer acetate, interferon beta-1a, interferon beta-1b, and peg-interferon beta-1a) or after other highly active DMTs (alemtuzumab, natalizumab, cladribine, fingolimod).

### 2.4. Persistence, Adherence, Healthcare Resource Utilization, and Costs

DMT discontinuation was defined as a switch to another DMT or complete discontinuation (i.e., no further record of medication initiation after 3 months from most recent refill of injectable and oral DMTs, after 3 months from natalizumab infusion, after 12 months from ocrelizumab infusion, after 18 months from year-1 dosing of cladribine or alemtuzumab, or after 36 months from year-2 dosing of cladribine or alemtuzumab) [2]. Persistence on treatment and related duration were calculated.

Adherence was estimated as the proportion of days covered (PDC). The PDC was calculated as the total days covered by the DMT divided by the length of the time period (expected refill/retreatment timing was calculated from the current regulatory indications); PDC ≥ 0.8 was considered adherent [2]. Considering that some DMTs have a low frequency of administration that would have caused too much variability in estimating adherence for 6 months (e.g., alemtuzumab, cladribine, ocrelizumab), we included patients with at least 12 months’ follow-up in the adherence analyses.

In Italy, all medications go through a multistage price definition before being made available on the market. The costs of DMTs are first negotiated by the Italian Agency of Medications (AIFA) and then by regional healthcare authorities (So.Re.Sa. for the Campania Region) for additional discounts. For comparability with other settings, the So.Re.Sa. provides regular updates of costs for all DMTs (https://www.soresa.it/pa/Pagine/Anagrafe/Farmaci-Emoderivati.aspx, accessed on 28 March 2024).

Healthcare resource utilization included MS-related hospital admissions (based on the main discharge diagnosis), from which we computed the annualized hospitalization rates (AHRs) [2]. We specifically included hospital admissions to identify the combination of MS and treatment issues (e.g., relapses and side effects), while we did not consider outpatients and day hospital admissions that could have been affected by the modality of administration (e.g., regular utilization for infusion DMTs).

The costs of MS-related hospital admissions and DMTs were directly derived from the So.Re.Sa. and inflated to the most recent values (2022) in order to avoid variations in price per unit of service over different years [2]. In particular, the DMT costs were reported at the time of collection, independently of the modality of administration.

Additional variables were age, sex, and comorbidities for patients with hospital discharge records, from which we computed the Charlson comorbidity index, assigning different weights to comorbidities reported with ICD codes [2,11].

### 2.5. Statistics

The study variables are presented as the mean (±standard deviation), number (percent), or median (range), as appropriate.

Differences between DMT groups were explored using Cox regression models (i.e., persistence) and linear regression models (i.e., adherence, AHR, costs), as appropriate. The covariates were age, sex, year of starting treatment (2018, 2019, 2020, 2021, and 2022), treatment duration, and adherence; statistical models were then run for the subgroup of patients with hospital discharge records, and the Charlson comorbidity index was included among the covariates.

The results were reported as the adjusted coefficient (Coeff), adjusted hazard ratio (HR), 95% confidence intervals (95%CI), and *p*-values, as appropriate. Statistical analyses were performed using Stata 15.0. The results were considered statistically significant at *p* < 0.05.

## 3. Results

**Figure 1:**
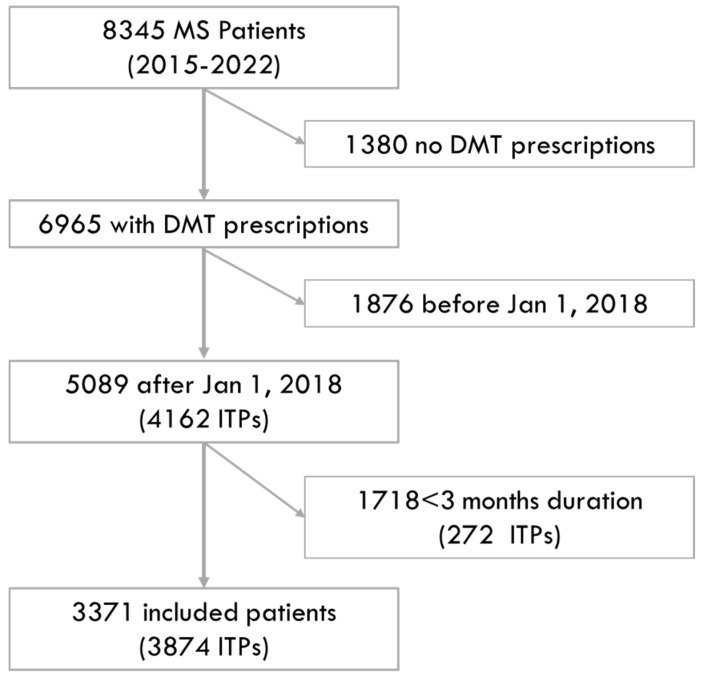
Study flow diagram. The figure shows the numbers of included and excluded patients, along with the reasons for exclusion.

From the population of people with MS in the Campania Region from 2015 to 2022 (n = 8345), we included 3371 individuals who commenced a DMT from 2018 to 2022, which corresponded to 3874 ITPs (with the same individual being treated with different DMTs within the study period). The reasons for exclusion are reported in Figure 1. The demographics, comorbidities and treatment features of the included patients (and the respective ITPs) are reported in Table 1.

Overall, we included 682 patients who were treated with ocrelizumab, corresponding to 682 ITPs. Looking at the administration modality, we included 480 ITPs with other DMTs administered via infusion (alemtuzumab, natalizumab), 1901 ITPs with oral DMTs (cladribine, fingolimod, teriflunomide, dimethyl fumarate), and 811 ITPs with injectable DMTs (glatiramer acetate, interferon beta-1a, interferon beta-1b, and peg-interferon beta-1a). Looking at the efficacy, we included 1080 ITPs with other highly active DMTs (alemtuzumab, natalizumab, cladribine, fingolimod) and 2112 with low-/medium-efficacy DMTs (teriflunomide, dimethyl fumarate, interferon beta-1a, interferon beta-1b, and peg-interferon beta-1a). The most frequently prescribed DMT was dimethyl fumarate (n = 815, 21.0%), followed by ocrelizumab (n = 682, 17.6%) (Table 1).

Most patients treated with ocrelizumab were newly diagnosed and drug naïve (n = 197, 28.8%), followed by patients who had previously been treated with fingolimod (n = 123), dimethyl fumarate (n = 87), teriflunomide (n = 72), natalizumab (n = 64), glatiramer-acetate (n = 52), alemtuzumab (n = 30), interferon beta1a (n = 29), interferon beta1b (n = 18), cladribine (n = 6), and peg-interferon beta1a (n = 4).

**Figure 2:**
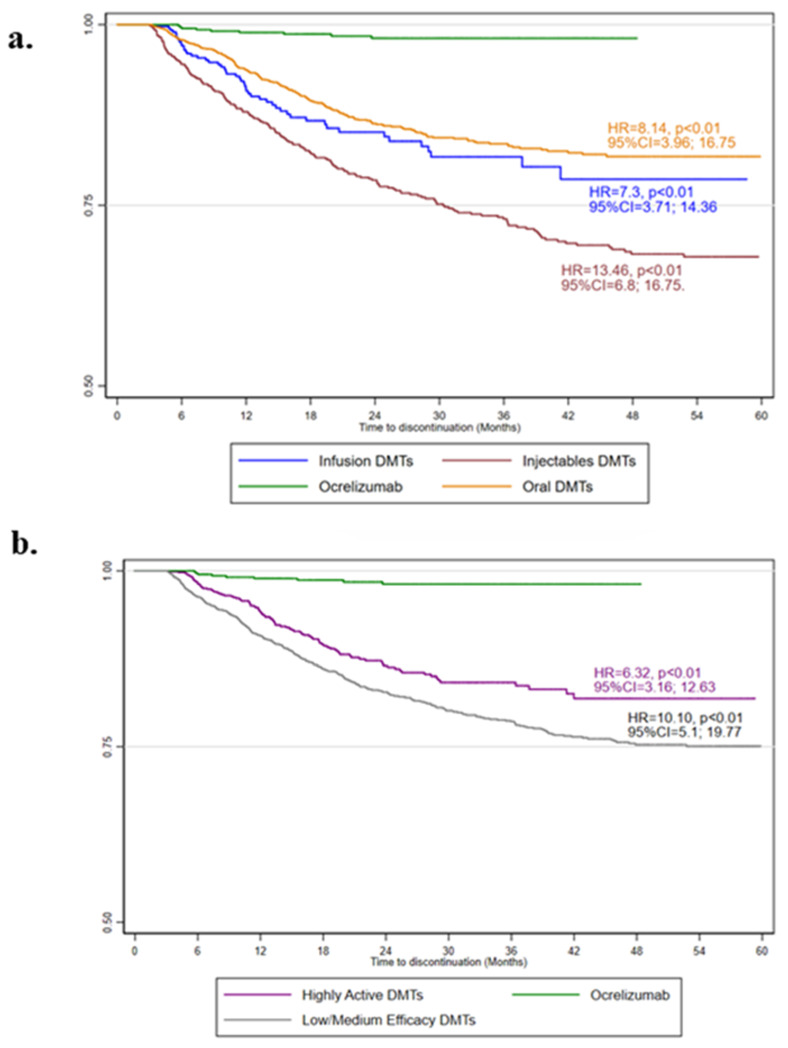
Kaplan–Meier estimates of treatment persistence. The adjusted hazard ratio (HR), 95% confidence intervals (95%CI), and *p*-values from Cox regression models evaluating the administration route (**a**) and clinical efficacy (**b**), with the age, sex, year of starting treatment (2018, 2019, 2020, 2021, or 2022), treatment duration, and adherence as covariates, are shown.

The ITP durations and the numbers of patients switching to other DMTs or completely discontinuing DMTs are reported in Table 2. A minority of ocrelizumab ITPs were discontinued (9 over 682) after 23.37 ± 11.28 months; in particular, two patients were switched to natalizumab, two were switched to dimethyl fumarate, 2 to cladribine, 2 to interferon beta1a, and 1 to glatiramer acetate. When compared with ocrelizumab, the risk of discontinuation was higher for other infusion (HR = 7.30; 95%CI = 3.71, 14.36; *p* < 0.01), oral (HR = 8.14; 95%CI = 3.96, 16.75; *p* < 0.01) and injectable DMTs (HR = 13.46; 95%CI = 6.80, 16.75; *p* < 0.01) (Figure 2a). Similarly, when compared with ocrelizumab, the risk of discontinuation was higher for other highly active (HR = 6.32; 95%CI = 3.16, 12.63; *p* < 0.01), and low-/medium-efficacy DMTs (HR = 10.10; 95%CI = 5.10, 19.77; *p* < 0.01) (Figure 2b). The results were also confirmed after adjusting for the Charlson comorbidity index.

Adherence to treatment is reported in Table 3. When compared with that for ocrelizumab, adherence (PDC) was lower for other infused (Coeff = −0.15; 95%CI = −0.18, −0.11; *p* < 0.01), oral (Coeff = −0.11; 95%CI = −0.14, −0.08; *p* < 0.01), and injectable DMTs (Coeff = −0.17; 95%CI = −0.20, −0.14; *p* < 0.01). When compared with that for ocrelizumab, adherence was lower for other highly active DMTs (Coeff = −0.07; 95%CI = −0.10, −0.04; *p* < 0.01) and low-/medium-efficacy DMTs (Coeff = −0.16; 95%CI = −0.19, −0.14; *p* < 0.01). The results were also confirmed after adjusting for the Charlson comorbidity index.

The healthcare resource utilization and costs are reported in Table 4. When compared with that for ocrelizumab, the AHR was similar for other infused (Coeff = 0.02; 95%CI = −0.01, 0.05; *p* = 0.13), oral (Coeff = 0.01; 95%CI = −0.03, 0.05; *p* = 0.61), and injectable DMTs (Coeff = −0.02; 95%CI = −0.05, 0.01; *p* = 0.06). When compared with that for ocrelizumab, the AHR was similar for other highly active (Coeff = −0.01; 95%CI = −0.03, 0.01; *p* = 0.35) and low-/medium-efficacy DMTs (Coeff = −0.02; 95%CI = −0.04, 0.01; *p* = 0.06). The results were also confirmed after adjusting for the Charlson comorbidity index.

When compared with those for ocrelizumab, the monthly costs for MS hospital admissions were similar for other infused DMTs (Coeff = 0.28; 95%CI = −11.66, 12.23; *p* = 0.96) but lower for oral (Coeff = −23.50; 95%CI = −45.17, −23.68; *p* < 0.01) and injectable DMTs (Coeff = −34.42; 95%CI = −32.45, −14.55; *p* < 0.01). When compared with those for ocrelizumab, the monthly costs for MS hospital admissions were similar for other highly active DMTs (Coeff = −8.93; 95%CI = −18.78, 0.92; *p* = 0.07) but lower for low-/medium-efficacy DMTs (Coeff = −29.76; 95%CI = −38.80, −20.71; *p* < 0.01). However, after adjusting for the Charlson comorbidity index, the monthly costs for MS hospital admissions were similar between ocrelizumab and other infused, oral, and injectable DMTs, as well as between ocrelizumab and other highly active and low-/medium-efficacy DMTs.

When compared with those for ocrelizumab, the monthly costs were similar to those of other infused DMTs (Coeff = −57.46; 95%CI = −138.56, 23.64; *p* = 0.16) but lower than those for oral (Coeff = −377.84; 95%CI = −428.24, −327.43; *p* < 0.01) and injectable DMTs (Coeff = −909.14; 95%CI = −969.69, −848.59; *p* < 0.01). When compared with those for ocrelizumab, the monthly costs were higher for other highly active DMTs (Coeff = 77.45; 95%CI = 29.36, 125.53; *p* < 0.01) but lower for low-/medium-efficacy DMTs (Coeff = −772.31; 95%CI = −816.95, −727.66; *p* < 0.01). The results were also confirmed after adjusting for the Charlson comorbidity index.

When considering only patients treated with ocrelizumab, the AHR was similar between ocrelizumab as a first-line DMT after low-/medium-efficacy DMTs (Coeff = −0.01; 95%CI = −0.05, 0.04; *p* = 0.84) and after other highly active DMTs (Coeff = 0.02; 95%CI = −0.02, 0.07; *p* = 0.34). Also, the monthly costs for MS hospital admissions were similar between ocrelizumab as a first-line DMT after low-/medium- efficacy DMTs (Coeff = −18.65; 95%CI = −47.26, 9.95; *p* = 0.20) and after other highly active DMTs (Coeff = −21.36; 95%CI = −51.05, 8.31; *p* = 0.15). The results were also confirmed after adjusting for the Charlson comorbidity index.

## 4. Discussion

In our 2018–2022 population-based study, we confirmed that ocrelizumab is among the most frequently prescribed DMTs in MS. When compared with our previous data (2018–2020) [2], thanks to the inclusion of about 3500 people with MS, we now showed that ocrelizumab prescriptions in incident cases of MS have risen further (28.8%), confirmed the favorable profile of ocrelizumab in terms of persistence, adherence, healthcare resource utilization, and costs, and explored the utilization of ocrelizumab in different treatment scenarios (e.g., DMT-naïve patients, comorbidities).

Ocrelizumab was among the most commonly prescribed DMTs in the Campania Region of Italy (17.6% of new DMT prescriptions from 2018 to 2022). Ocrelizumab was preferred as a first-line DMT in 28.8% of cases and was used within escalation strategies from platform DMTs in 38.5% of patients. The remaining patients (32.7%) received ocrelizumab following other high-efficacy DMTs due to a combination of tolerability and efficacy issues [12]. As such, the increasing utilization of ocrelizumab possibly reflects unmet needs in MS, especially in the case of progressive aspects [13,14]. This prescription pattern is in line with that found in studies conducted within the same time frame in the US [15] and Australia [16].

Ocrelizumab was associated with the highest rates of persistence and adherence among the DMTs, with 1.3% patients being discontinued and 92.1% being fully adherent, suggesting optimal effectiveness and safety [2]. Ocrelizumab was already proven to have high persistence rates in previous studies [17,18,19,20], with efficacy and safety issues being the most common causes of the few cases of discontinuation [17,20]. Indeed, looking at recent registry data, relapses, disability progression, and MRI activity are expected to occur in a minority of patients treated with ocrelizumab [21,22,23,24,25]. Notably, looking at demographics, ocrelizumab was used in much more complex populations (i.e., older age, higher comorbidity burden) when compared with other high-efficacy DMTs, as already described in some previous studies [17,21,22], where effectiveness was not granted. In particular, the effectiveness of ocrelizumab for relapses was also recently confirmed in people above 60 years of age with MS, though this was balanced by the low relapse rate and the lack of statistical significance for disability progression [26]. Also, side effects were reported by 10% of patients treated with ocrelizumab, and they mostly consisted of mild infusion-related reactions and infections [17,22,24] and were independent from age [21]. Taken together, our data on the high persistence and adherence to ocrelizumab might reflect continuing and satisfactory balance between efficacy and safety over 5 years of follow-up.

We found high rates of adherence to ocrelizumab, suggesting that infusions were scheduled every 6 months for most patients. Extended-interval dosing of ocrelizumab was preliminary described within the COVID-19-related re-organization of healthcare services [27,28,29] and was then further studied in other retrospective studies that included patients who were given extended intervals due to different reasons [30]. Interestingly, the latter could be the case in 7.9% of our population treated with ocrelizumab, where the infusion interval was >1 month longer than the conventional 6 months. In any case, this percentage of patients with an extended infusion interval for ocrelizumab was relatively low, suggesting that there was no specific need to delay infusions in most cases.

Our study confirmed that ocrelizumab has lower direct treatment costs than those of other highly active DMTs [2], thus further reinforcing its cost-saving value or, at least, cost effectiveness [14]. This is partly due to the overall costs of ocrelizumab but also relates to the modalities of utilization, including in MS patients in the early stages of the disease and with a higher comorbidity burden, where other DMTs do not perform equally well. In particular, we showed that ocrelizumab was associated with a similar probability of MS-related hospital admissions and costs when compared with other similar or less effective DMTs, especially when accounting for comorbidities. In keeping with this, in a previous US claims study that included 189 patients treated with ocrelizumab, alemtuzumab, or natalizumab for 1 year, the authors showed reduced costs of ocrelizumab treatment and related procedures [31]. Also, Rog and colleagues showed that ocrelizumab was associated with lower administration and monitoring burden for healthcare professionals when compared with other infusion DMTs [32]. Taking these results together, ocrelizumab was less expensive than other high-efficacy DMTs and had similar patterns of MS hospital admissions and related costs to those of other DMTs, which mostly reflected disease or treatment complications [11]. Looking at patients treated with ocrelizumab, we found very low numbers of hospital admissions (and lack of statistical significance) independently from its use as a first-line drug after low-/medium-efficacy DMTs and after highly-active DMTs. In a previous study, Geiger and colleagues found lower hospitalization rates in patients treated with ocrelizumab as a first-line DMT when compared with its use as a second-line DMT, thus suggesting that the most cost-effective utilization of ocrelizumab was in treatment-naïve patients [12]. In our study, we also analyzed hospitalization rates depending on the use of ocrelizumab as a first-line or switch treatment but found 10-fold-lower hospitalization rates, as often described in European studies when compared with those in the US [11,12,33], and there was a lack of statistical significance. The small number of hospital admissions also did not allow any statistical analyses related to the main driver of the admission (i.e., relapse, infection, etc.).

The limitations of our study include the generalizability of our results, since we only included patients from a specific Italian region. However, our cohort had a similar distribution (e.g., age, DMT use) to that in other international studies [16,21,22,34] and, hence, may reflect the general MS population treated with ocrelizumab. Our study also holds limitations derived from the use of routinely collected healthcare data, including the definition of MS-related hospital admission based on the primary diagnosis, which could be biased by physicians’ perspectives, as well as the definition of adherence based on DMT infusion or refill, which could be biased by the fact that oral and injectable DMTs are collected but not actually taken. The lack of clinical data does not allow inference on potential changes in societal costs from different effects on relapses and disability for DMTs or on specific treatment strategies, including infection risk minimization in elderly or at-risk populations (e.g., those with low levels of IgG) [35]. Additional DMTs, including ofatumumab, ozanimod, ponesimod, and siponimod, were progressively approved in the Campania Region in 2022 and, thus, were not included in our study due to the short follow-up.

## 5. Conclusions

In conclusion, we confirmed previous results on the higher persistence and adherence rates of ocrelizumab when compared with other DMTs of similar efficacy and modes of administration. We also showed that ocrelizumab is less expensive than other high-efficacy DMTs while possibly being equally effective based on indirect measures from routinely collected healthcare data (i.e., hospital admissions and related costs).

## Figures and Tables

**Table 1:** Demographics, comorbidities, and treatment features.

DMT	Patients	ITPs	Age	Females	Charlson Comorbidity Index		
	(n)		(Years)	(n)	0	1–2	≥3
Ocrelizumab	682	682	46.46 ± 11.29	384	556	18	2
Alemtuzumab	41	41	34.63 ± 7.97	30	30	-	-
Natalizumab	438	439	34.78 ± 12.05	297	323	4	2
Cladribine	141	141	41.22 ± 12.09	98	87	3	-
Fingolimod	459	459	39.26 ± 12.03	290	243	-	-
Teriflunomide	483	486	50.06 ± 11.59	316	156	10	1
Dimethyl fumarate	814	815	38.90 ± 11.91	566	247	3	2
Interferon beta1a im	95	95	48.94 ± 13.94	64	18	2	-
Interferon beta1b	69	70	52.79 ± 10.38	41	10	-	-
Glatiramer acetate	267	267	46.29 ± 12.04	184	57	4	-
Peg-interferon beta1a	99	99	38.90 ± 13.85	76	19	1	-
Interferon beta1a sc	280	280	40.89 ± 12.80	214	84	1	-

**Table 2:** Treatment duration.

DMT	ITP Duration (Months)		Switch to Other DMT	Complete DMT Discontinuation
	Mean ± SD	Median (IQR)		
Ocrelizumab	23.37 ± 11.28	(13–31)	8	1
Alemtuzumab	12.98 ± 2.80	(11–14)	5	4
Natalizumab	20.29 ± 13.94	(8–31)	42	7
Cladribine	12.47 ± 1.44	(12–13)	7	-
Fingolimod	25.60 ± 13.48	(13–36)	38	9
Teriflunomide	26.45 ± 17.01	(12–39)	44	16
Dimethyl fumarate	28.86 ± 16.71	(14–42)	100	14
Interferon beta1a im	35.01 ± 19.62	(14–55)	12	2
Interferon beta1b	34.14 ± 20.36	(12–55)	12	3
Glatiramer acetate	29.92 ± 18.91	(10–46)	55	17
Peg-interferon beta1a	24.95 ± 16.73	(14–38)	24	2
Interferon beta1a sc	31.73 ± 19.16	(12–51)	70	10

The table shows the mean (±standard deviation (SD)) and median (and interquartile range (IQR)) of the duration of IPTs and the number of patients that were switched to other DMTs or were completely discontinued from DMTs.

**Table 3:** Adherence.

DMT	PDC	PDC > 0.8	
Ocrelizumab	1.03 ± 0.24	628/682	92.0%
Alemtuzumab	1.02 ± 0.08	15/15	100.0%
Natalizumab	0.95 ± 0.15	373/438	85.1%
Cladribine	1.10 ± 0.13	141/141	100.0%
Fingolimod	0.91 ± 0.25	326/452	72.1%
Teriflunomide	0.86 ± 0.34	264/433	60.9%
Dimethyl fumarate	0.90 ± 0.28	501/727	68.9%
Interferon beta1a im	0.91 ± 0.32	55/79	69.6%
Interferon beta1b	0.89 ± 0.34	44/67	65.6%
Glatiramer acetate	0.91 ± 0.31	168/254	66.1%
Peg-interferon beta1a	0.99 ± 0.29	72/93	77.4%
Interferon beta1a sc	0.91 ± 0.28	183/267	68.5%

The table shows the proportion of days covered (PDC) for each DMT, which was calculated as the total days covered during 1 year divided by 365 days of follow-up (according to the current regulatory indications), for each ITP. The number and percentage of patients with PDC above 80% are also reported.

**Table 4:** Healthcare resource utilization and costs.

DMT	MS Hospital Admissions		AHR	DMT Costs
	Number	Costs (EUR/Month)		(EUR/Month)
Ocrelizumab	65	46.93 ± 151.34	0.06 ± 0.25	1670.34 ± 436.24
*First-line DMT*	13	62.27 ± 220.04	0.04 ± 0.23	
*After low-/medium-efficacy DMTs*	30	42.14 ± 115.75	0.05 ± 0.21	
*After other highly active DMTs*	22	38.99 ± 107.03	0.06 ± 0.29	
Alemtuzumab	5	31.97 ± 46.87	0.12 ± 0.41	3442.94 ± 725.42
Natalizumab	34	45.48 ± 165.29	0.06 ± 0.45	1632.86 ± 424.86
Cladribine	3	38.67 ± 39.71	0.02 ± 0.13	3048.97 ± 804.22
Fingolimod	10	27.15 ± 52.96	0.01 ± 0.10	1523.74 ± 427.02
Teriflunomide	32	22.35 ± 97.54	0.03 ± 0.19	796.30 ± 181.49
Dimethyl fumarate	36	14.30 ± 43.53	0.03 ± 0.17	1041.90 ± 266.66
Interferon beta1a im	5	3.63 ± 12.49	0.01 ± 0.07	797.98 ± 299.36
Interferon beta1b	3	9.43 ± 72.49	0.01 ± 0.06	473.48 ± 153.09
Glatiramer acetate	23	9.99 ± 51.37	0.03 ± 0.16	580.12 ± 287.45
Peg-interferon beta1a	2	8.76 ± 49.50	0.05 ± 0.40	741.37 ± 239.00
Interferon beta1a sc	10	10.07 ± 28.38	0.02 ± 0.15	864.14 ± 430.87

The table shows the number of MS-related hospital admissions and related costs. Annualized hospitalization rates (AHRs) for MS-related admissions are also reported. For ocrelizumab, the data are divided into patients receiving ocrelizumab as a first-line DMT after low-/medium-efficacy DMTs or after other highly active DMTs. The costs are based on actual DMT refills/administrations per patient and refer to a month of 30.5 days.

## Data Availability

Data are available upon request to the Regional Healthcare Society (So.Re.Sa—www.soresa.it).
